# Spontaneous tumor lysis syndrome in an adult with alveolar rhabdomyosarcoma: a challenging diagnosis

**DOI:** 10.1093/omcr/omae043

**Published:** 2024-05-20

**Authors:** Arein A Abufara, Mohammad I Alsahouri, Qusai A Alsalah, Hasan Arafat, Ahmad G Hammouri, Bashir Abu Aqeel

**Affiliations:** Faculty of Medicine, Palestine Polytechnic University, Hebron, Palestine; Faculty of Medicine, Palestine Polytechnic University, Hebron, Palestine; Faculty of Medicine, Palestine Polytechnic University, Hebron, Palestine; Department of Internal Medicine, Augusta Victoria Hospital, Jerusalem, Palestine; Radiology Department, Al-Ahli Hospital, Hebron, Palestine; Cancer Care Center, Augusta Victoria Hospital, Jerusalem, Palestine

## Abstract

Tumor lysis syndrome (TLS) is an oncological emergency characterized by metabolic and electrolyte imbalances associated with the rapid destruction of tumor cells. It is commonly recognized when cytotoxic treatment for hematological malignancies is initiated. Spontaneous TLS with solid tumors like rhabdomyosarcoma (RMS) is exceedingly rare. It has been noted that the highest incidence of this tumor occurs in individuals under the age of 20 years, with an incidence rate of 4.4 cases per 1 million. Here, we present the case of a 22-year-old male who presented with spontaneous clinical TLS. A computed tomography (CT) scan revealed a large pelvic mass, diffuse lymphadenopathy, and infiltration of the ocular muscles. Subsequently, a biopsy was conducted, and the histopathological results indicated alveolar rhabdomyosarcoma. Our literature review revealed five cases of spontaneous TLS caused by RMS, with our patient being the only adult among all published cases.

## INTRODUCTION

Tumor lysis syndrome (TLS) is a life-threatening oncological emergency that arises from the rapid destruction of tumor cells, leading to the rapid release of several intracellular metabolites and the emergence of hyperphosphatemia, hyperkalemia, and hyperuricemia [1]. The most severe symptoms of these electrolyte imbalances are acute renal failure and cardiac arrhythmia [2]. TLS is most frequently seen in hematological neoplasms that are chemosensitive [1]. Infrequently, TLS may also happen spontaneously [2]. In patients with solid tumors, TLS is extremely uncommon [3].

Rhabdomyosarcoma (RMS) is a tumor that typically affects children and adolescents and with most commonly location in the head and neck [4]. It is uncommon in adults, representing 3% of soft tissue sarcomas and < 1% of all solid tumors in adult malignancies [5]. Our study focuses on a 22-year-old male who presented with spontaneous clinical TLS attributed to RMS in the prostate. This report aims to shed light on this rare entity and emphasizes the importance of not underestimating the risk of developing spontaneous TLS in individuals with RMS.

## CASE PRESENTATION

A 22-year-old male presented with complaints of lower abdominal and pelvic pain, left eye swelling, and hematuria. Imaging revealed an enlarged prostate invading surrounding structures and multiple enlarged lymph nodes. A true-cut biopsy identified a malignant small round blue cell tumor. Consequently, the patient was referred to our oncology department for further evaluation and treatment. Upon admission, the patient appeared pale, with a blood pressure of 140/90 mmHg and a heart rate of 107 bpm. Physical examination revealed left eye swelling with ecchymosis, conjunctival bleeding in the right eye, palpable hard mass at the left neck’s mandibular angle, bilateral axillary lymphadenopathy, and distended abdomen with suprapubic fullness. An electrocardiogram showed sinus tachycardia. The patient had an Eastern Cooperative Oncology Group Performance Status (ECOG-PS) of four.

Laboratory analysis revealed anemia, severe thrombocytopenia, hyperphosphatemia, hyperuricemia, severe hyponatremia, high-normal potassium, normal calcium, elevated creatinine, blood urea nitrogen, and lactate dehydrogenase (Table 1).

**Table 1:** Patient’s laboratory data on admission

Laboratory test and Tumor marker	values	Reference range
Hemoglobin	7.6 g/dl	13.50–18 g/dl
platelet	4.13 × 10^3/ul	150–450 × 10^3^/ul
Phosphorous	8.25 mg/dl	2.50–4.50 mg/dl
Calcium	8.94 mg/dl	8.60–10.30 mg/dl
Potassium	5.27 mmol/l	3.50–5.30 mmol/l
Sodium	118 mmol/l	136–145 mmol/l
Albumin	3.17 g/dl	3.7–5.2 g/dl
Uric Acid	15.2 mg/dl	3.40–7 mg/dl
Blood Urea Nitrogen	63.3 mg/dl	6–20 mg/dl
creatinine	4.14 mg/dl	0.70–1.20 mg/dl
Beta-Human Chorionic Gonadotropins (Beta-hCG)	0.203 mIU/ml	0–5 mIU/ml
Carcinoembryonic antigen (CEA)	1.22 ng/ml	0–2.9 ng/ml
Prostate-specific antigen (PSA)	0.179 ng/ml	0–4 ng/ml
Carbohydrate antigen 19-9 (CA19-9)	36.16 u/ml	0–27 u/ml
Alpha-fetoprotein (AFP)	4.92 ng/ml	0–15 ng/ml
Lactate dehydrogenase (LDH)	5666 u/l	135–225 u/l

Following the Cairo-Bishop definition, the patient was diagnosed with tumor lysis syndrome (TLS). The patient was admitted to the intensive care unit, he received cautious IV hydration, hypertonic saline for severe hyponatremia, sevelamer for hyperphosphatemia, and Rasburicase for hyperuricemia.

**Figure 1:**
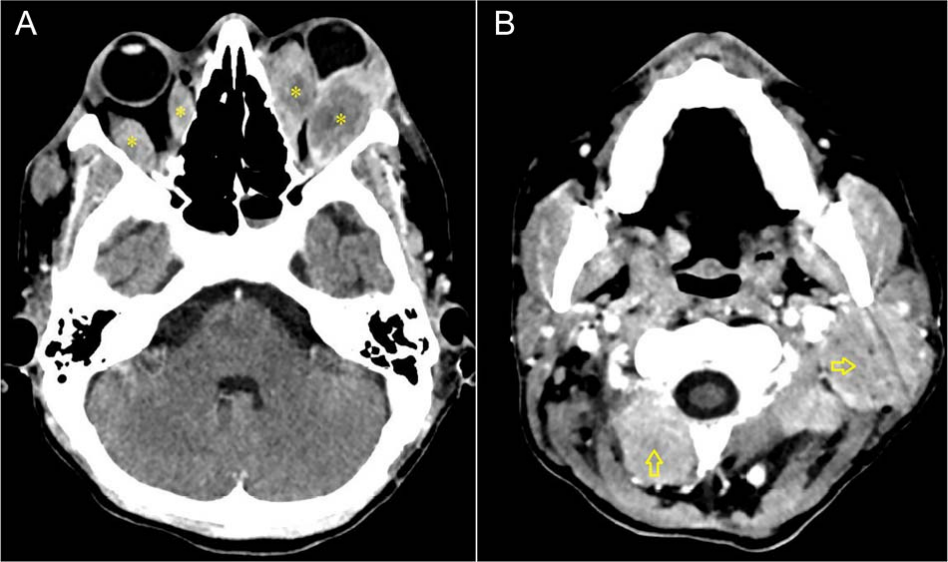
Selected axial cut of the brain CT scan for our patient (**A**) demonstrates a bilateral asymmetrical fusiform enlargement of the extra-ocular orbital muscles (Asterisks) most appreciated at the lateral rectus muscles and more at the left side, relatively sparing the anterior tendon. Associated with exophthalmos. Neck CT scan selected axial cut (**B**) Shows bilateral enlarged cervical lymph nodes (Arrows).

**Figure 2:**
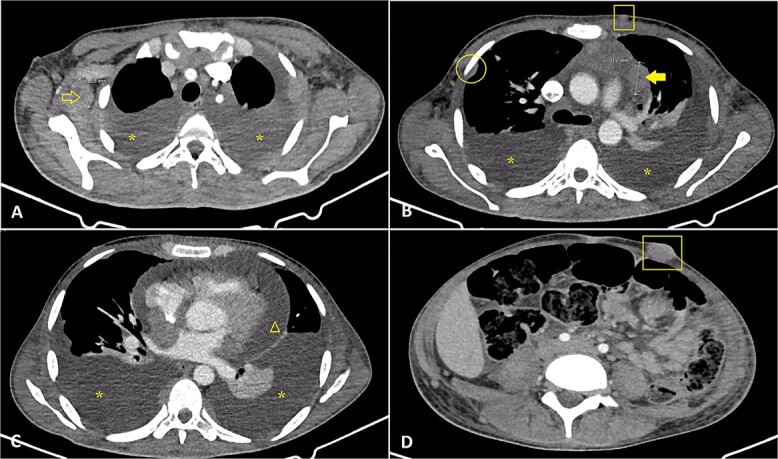
Selected axial cuts of a chest and abdomen CT scan were done for the patient, showing moderate bilateral pleural effusions (Asterisks) (**A**, **B**, and **C**) and pericardial effusion (Triangular) (**C**). In addition, pleural-based nodules are noted (Circle) (**B**), associated with multiple enlarged mediastinal (Solid arrow) and bilateral axillary (Open arrow) lymph nodes (**A**, **B**). A few subcutaneous enhancing nodules are also noted (Squares) (**B**, **D**). These features are related to widespread metastatic disease.

A CT scan without contrast revealed a large pelvic mass with extensive local advancement that exerted compression on both the rectum and bladder. Diffuse lymphadenopathy was observed, and infiltration of the ocular muscles in the left eye was identified (Figs 1 and 2).

**Figure 3:**
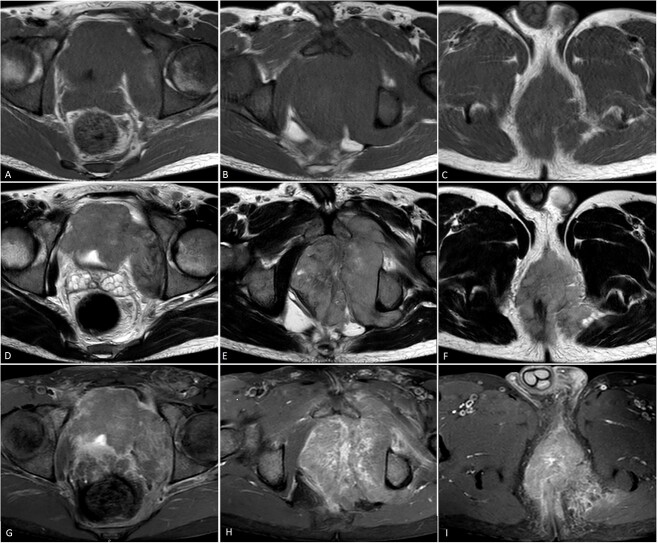
Pelvic MRI for the patient showing a huge pelvic mass, extending through the left obturator canal invading the left obturator externus muscle (**E** and **H**). The mass measures up to 12 cm in its greatest axial dimension and is noted to be iso-intense on T1W images (**A**–**C**), heterogeneously hyper-intense on T2W images (**D**–**F**), and displaying heterogeneous enhancement on Fat saturated T1W images with Contrast medium (**G–I**). Multiple enlarged pelvic lymph nodes were seen (Not shown here).

**Figure 4:**
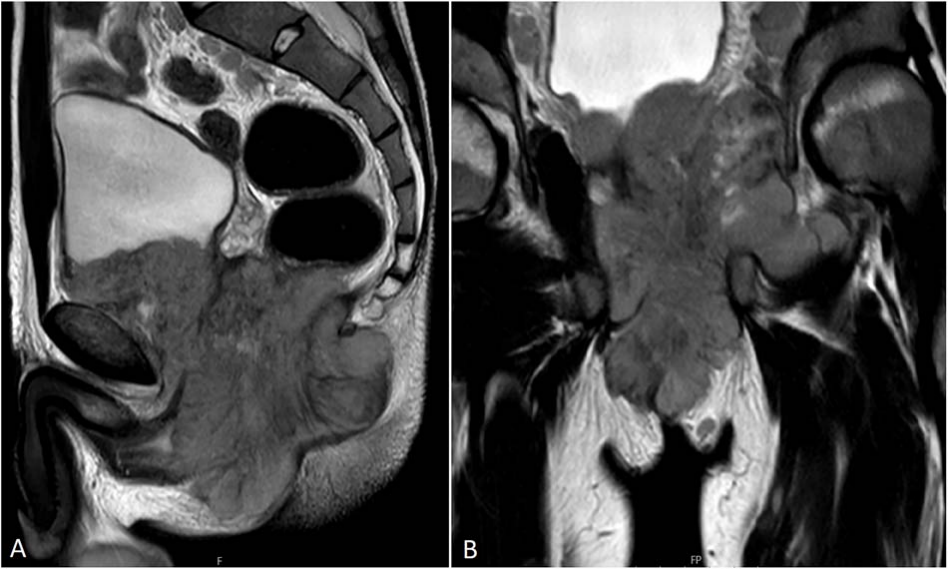
Selected sagittal (**A**) and coronal (**B**) T2W Pelvic MRI images better demonstrate the extension of the mass, which is noted to invade the UB base superiorly. Multiple enlarged pelvic lymph nodes are also seen.

MRI showed a massive pelvic mass measuring approximately 12 cm in its greatest dimension, invading the left obturator externus muscle and bladder (Figs 3 and 4).

**Figure 5:**
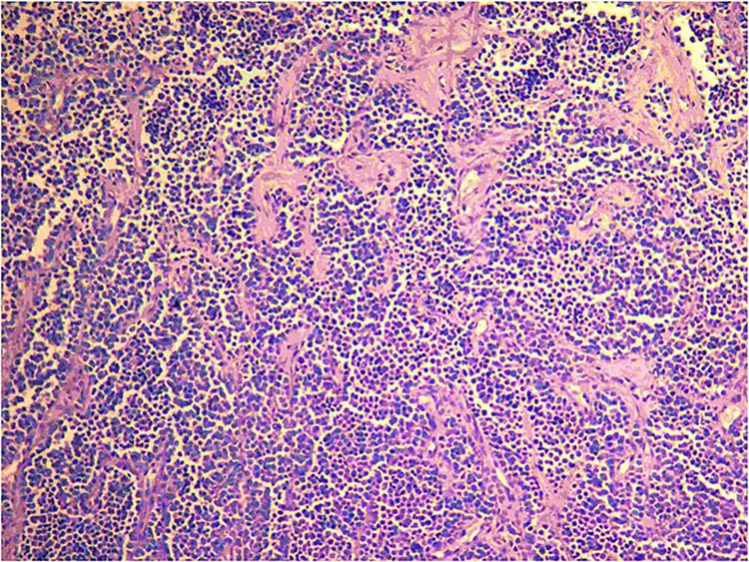
Histopathological features of the mass: The tumor cells are composed of larger, uniformly round to polygonal cells with a variable number of rhabdomyoblasts, alveolar subtype. Multinucleated tumor giant cells with wreath-like nuclei are seen in addition to a high index of mitoses (H&E staining; magnification, ×20).

**Figure 6:**
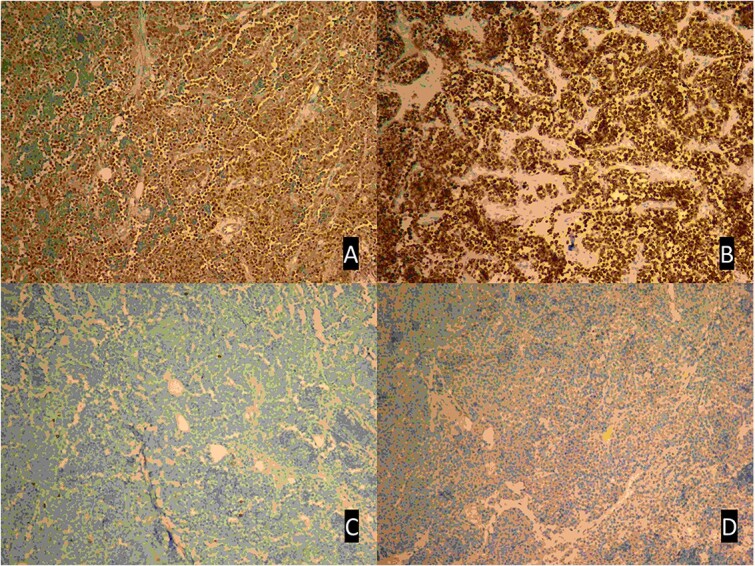
The Immunohistochemical studies of the tumor cells revealed intense myogenin expression (**A**) and positive immunostaining for desmin (**B**). However, the results were negative for CD45 (**C**) and TdT (**D**). Magnification: ×20.

**Figure 7:**
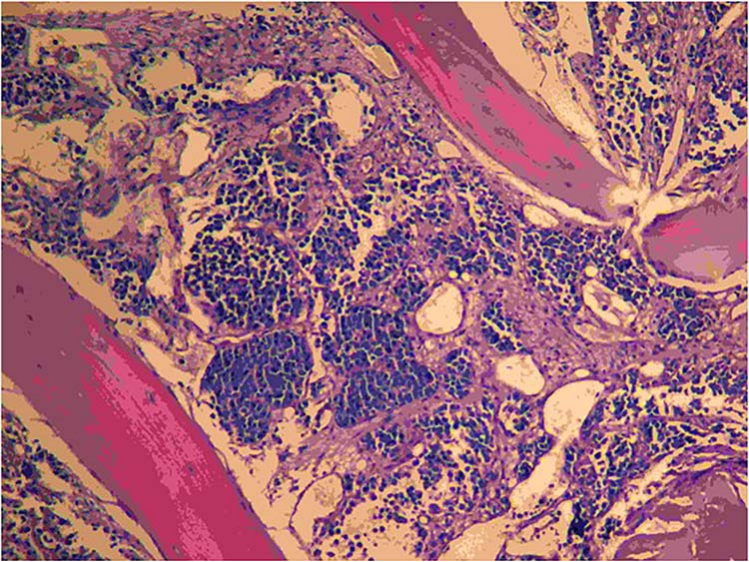
The bone marrow trephine biopsy picture here shows the effacement of the marrow spaces by a uniform cell population consisting of cells with a high nuclear-to-cytoplasmic ratio. The cells are arranged in variably sized nests separated by fibrous tissue septa. In places, the cells appear loosely dispersed, mimicking a pulmonary alveolar pattern, hence the name.

**Figure 8:**
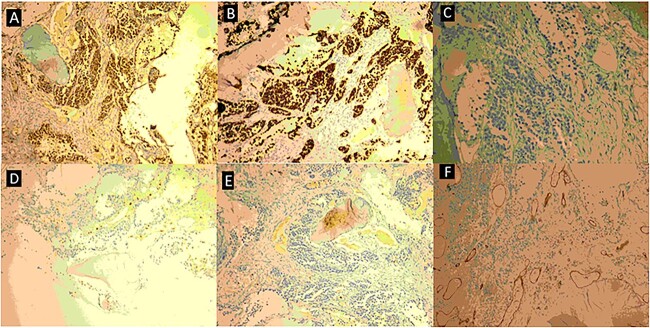
Immunostaining pattern of bone marrow cells demonstrates intense myogenin expression (**A**) and positive immunostaining for desmin (**B**), while they are negative for Ckit (**C**), CD3 (**D**), CD20 (**E**), and CD34 (**F**), it was also negative for Synaptophysin, Fli-1, TdT, and CK-pan (not shown here). These findings were suggestive of metastatic alveolar RMS.

Histopathological review of the paraffin blocks was consistent with rhabdomyosarcoma, mostly the alveolar subtype (Fig. 5). Immunohistochemistry demonstrated positive myogenin and desmin staining (Fig. 6). Considering lymphadenopathy and high LDH, a second primary malignancy, possibly lymphoma, couldn’t be ruled out. Diffuse lymphadenopathy, bicytopenia, and spontaneous TLS suggested hematologic malignancy. Consequently, a bone marrow biopsy revealed rhabdomyosarcoma infiltration with a staining pattern similar to the provided paraffin blocks (Figs 7 and 8). Excisional lymph node and abdominal wall nodule biopsies confirmed the presence of rhabdomyosarcoma.

Due to the patient’s persistent TLS and bicytopenia, as well as unresponsive hematuria and anemia despite medical management, a decision was made to initiate chemotherapy promptly. The patient was started on IE protocol applied in our hospital which includes: (ifosfamide 1800 mg/m2 for five days, etoposide 100 mg/m2 for five days, and mesna 600 mg/m2 three times per day for five days, in addition to filgrastim 300 mcg subcutaneously 24 h after chemotherapy for ten days) with 50% of the dose. On day three of the protocol, the patient was noticed to be sleepier, and less active with new hallucinations, giving an impression of ifosfamide-induced encephalopathy. Ifosfamide was omitted, and methylene blue and thiamine were commenced, with a pronounced response.

Left eye swelling with ecchymosis and infiltration of the ocular muscles, as revealed by a CT scan, indicated evidence of metastatic retroorbital lesions. Consequently, the patient underwent 30 grays over 5 fractions of palliative radiotherapy using a three-dimensional technique targeting the left orbit. This treatment resulted in good local control of symptoms and reduced swelling.

The patient hemoglobin level and platelet count started to increase within seven days of finishing the chemotherapy protocol, his hemoglobin level remained around 8.5, no longer in need of blood transfusion, while his platelet count reached a peak of 65 × 10^3^/ul, and remained above 50 × 10^3^/ul. He was scheduled for 4–6 cycles before assessing response via PET scan.

## DISCUSSION

Tumor Lysis Syndrome (TLS) is an oncological emergency that results from tumor cells destruction and is commonly linked with hematological malignancies when cytotoxic agents are administered [1]. In the Cairo-Bishop classification, TLS is classified as laboratory or clinical TLS (Table 2) [6].

**Table 2:** Cairo–Bishop definition of laboratory and clinical tumor lysis syndrome [6]

Laboratory TLS	Clinical TLS
Presence of ≥2 of the following around the period of treatment initiation (including 3 days prior and 7 days after): K^+^ > 6.0 mEq/l or 25% increase from baseline PO_4_^3−^ > 4.5 mg/dl (1.45 mmol/l) or 25% increase from baseline Uric acid >8.0 mg/dl (476 micromol/l) or 25% increase from baseline Corrected calcium < 7.0 mg/dl (1.75 mmol/l) or ionized Ca^2+^ < 4.5 mg/dl (1.12 mmol/l) or 25% decrease from baseline	Presence of laboratory TLS plus ≥1 of the following: Creatinine levels ≥1.5 × the upper reference range Seizures Cardiac arrhythmias

Generally, solid tumors in adults do not exhibit enough sensitivity to chemotherapy to trigger TLS [1]. However, TLS occurrence in individuals with solid tumors is rare, yet it is linked with unfavorable clinical outcomes and an elevated mortality rate, particularly in the presence of rare tumor types like sarcomas or in instances of spontaneous onset [3]. Rhabdomyosarcoma (RMS) is a prevalent cancer that typically occurs during childhood and adolescence, accounting for half of all soft tissue sarcomas. The highest occurrence is observed in individuals under the age of 20, with an incidence rate of 4.4 cases per 1 million [7], The two predominant forms of RMS are alveolar (ARMS) and embryonal (EMRS), with ARMS being more prevalent among adults [5, 7]. ARMS involving lymph nodes is a significant and difficult diagnostic issue, particularly in cases where patients exhibit laboratory or clinical evidence of acute TLS [4]. The most common location for RMS is the head and neck area among children, while in adults, it predominantly affects the extremities [7]. This diagnosis is exceptionally rare, with only five cases of spontaneous TLS due to RMS identified in our literature review [1, 4, 8, 9] (Table 3).

**Table 3:** A summary of the characteristics of worldwide five cases of spontaneous Tumor lysis syndrome (TLS) due to Rhabdomyosarcoma (RMS) that have been published previously, along with our case

Authors	Age/Gender	Type of TLS	Primary tumor	Histology	Metastatic	Outcome
Jani et al. [9]	16/M	CTLS	Not detected	ARMS	Bone marrow, bone, subcutaneous tissue, lungs, pleura, and mediastinal and hilar lymph nodes.	Death.
Bien et al. [8]	14/M	LTLS	Not detected	EMRS	Bone marrow, pleura, peritoneum, Lymph node	Survived
Bien et al. [8]	14.5/F	LTLS	Parietal region	Unclassified RMS	Bone marrow	Survived
Watanabe et al. [1]	16/M	LTLS	Prostate	ARMS	Bone marrow, Lymph node, bladder wall	Survived
Patiroglu et al. [4]	14/M	CTLS	Not detected	ARMS	Bone marrow, Lymph node	Death
Our case	22/M	CTLS	Prostate	ARMS	Pelvic, ocular muscles in the left eye, Lymph node	Death

M: Male, F: Female, CTLS: clinical tumor lysis syndrome, LTLS: laboratory tumor lysis syndrome, ARMS: alveolar Rhabdomyosarcoma, EMRS: embryonal Rhabdomyosarcoma.

In 2008, an international panel on TLS consensus formulated a model for risk classification and provided prophylactic recommendations. The majority of solid tumors fall into the low-risk category, including RMS. Nonetheless, bulky chemosensitive tumors, such as small cell lung cancers, germ cell tumors, and neuroblastoma, are linked to an intermediate risk of TLS. Additionally, they are categorized as high risk if the patient exhibits renal dysfunction and/or laboratory evidence of TLS [10].

Due to the possible seriousness of complications arising from TLS, it is crucial to take preventive measures in high-risk individuals and initiate treatment if symptoms emerge. Hydration is the major method for both prevention and therapy of TLS [1, 10]. For low-risk individuals, oral hydration is suggested, with careful fluid balance monitoring. Intermediate and high-risk patients, as well as those with established TLS, should receive aggressive IV hydration with isotonic crystalloids. Regular electrolyte monitoring is essential, with specific therapies for hyperkalemia, hyperphosphatemia, and symptomatic hypocalcemia [10].

Allopurinol is recommended for prophylaxis in low to intermediate-risk hyperuricemia patients, whereas Rasburicase is indicated for treatment and prophylaxis in high-risk cases [10].

Allopurinol functions by inhibiting xanthine oxidase, thereby impeding the production of further uric acid. In contrast, Rasburicase converts uric acid into the soluble compound allantoin. Allantoin exhibits a solubility in urine that is 5 to 10 times higher than that of uric acid [1].

## CONCLUSION

While TLS is most commonly associated with hematological malignancies, our experience demonstrates its occurrence in solid tumors, albeit less frequently. Our report highlights the importance of recognizing TLS as a potential complication of RMS. Clinicians should maintain a high index of suspicion, even in rare scenarios, to ensure timely intervention and optimal patient outcomes. Furthermore, this case prompts a reevaluation of the risk stratification and management protocols for RMS, as we recommend treating RMS similarly to neuroblastoma due to its rapid growth and high chemosensitivity. This implies upgrading RMS from low risk to intermediate risk to address the potential development of TLS.

## Data Availability

The data used to support the findings of this study are included in the article.
